# Rate and Causes of Unplanned Hospital Returns within 60 Days following Head and Neck Surgery

**DOI:** 10.1055/s-0044-1779433

**Published:** 2024-02-16

**Authors:** Mazin Merdad, Abdulsalam Alqutub, Ahmed Mogharbel, Abdullah Farid, Abdullah Bayazed, Abdulaziz Alghamdi, Yazeed Albogami, Rayan Alshehri, Majed N. Alnefaie, Hanin A. Alamoudi

**Affiliations:** 1 Department of Otolaryngology, Head and Neck Surgery, King Abdulaziz University Hospital, King Abdulaziz University, Jeddah, Kingdom of Saudi Arabia; 2 Department of Otolaryngology, Head and Neck Surgery, Faculty of Medicine, King Abdulaziz University, Jeddah, Kingdom of Saudi Arabia; 3 Department of Otolaryngology, Head and Neck Surgery, King Fahd Armed Forces Hospital, Jeddah, Kingdom of Saudi Arabia

**Keywords:** patient readmission, head and neck neoplasm, surgery, otolaryngology, comorbidity

## Abstract

**Introduction** Unplanned hospital returns are frequent and may be preventable.

**Objective** To comprehend the reasons for unplanned hospital readmission and return to the Outpatient Department (OPD) and Emergency Department (ED) within 60 days after discharge following head and neck surgery (HNS) at a tertiary care center in Saudi Arabia.

**Methods** In the present retrospective study, the medical records of all patients who underwent HNS for benign and malignant conditions between January 2015 and June 2022 were reviewed in terms of demographic data, comorbidities, and reasons for hospital return.

**Results** Out of 1,030 cases, 119 (11.55%) returned to the hospital within 60 days after discharge, 19 of which (1.84%) were readmitted. In total, 90 (8.74%) patients returned to the OPD, and 29 (2.82%), to the ED. The common reasons for readmission included infections (26.32%) and neurological symptoms (21.05%). For OPD visits, the common causes were hematoma (20%) and neurological symptoms (14.44%). For ED returns, the frequent causes were neurological symptoms (20.69%) and equipment issues (17.24%). Compared with nonreadmitted patients, readmitted patients had a higher preoperative baseline health burden when examined using the American Society of Anesthesiologists (ASA) score ( *p* = 0.004) and the Cumulative Illness Rating Scale (CIRS; *p* = 0.002).

**Conclusion** The 60-day rates of unplanned hospital return to the OPD and ED were of 8.74% and 2.82% respectively, and 1.84% of the patients were readmitted. Hematoma, infections, and neurological symptoms were common causes. Addressing the common reasons may be beneficial to decrease postoperative hospital visits.

## Introduction

Unplanned postoperative hospital returns are frequent, costly, and perhaps avoidable with careful planning and patient education. 1 2 3 Many institutions identify the rate of unplanned hospital revisits as an indicator of the quality of care. 4 Thus, decreasing hospital revisits is increasingly crucial for clinicians, hospitals, and policymakers. 5 Head and neck surgery (HNS), particularly oncologic HNS, comprises multiple-step procedures, including resections, vascularized tissue reconstruction, and extensive neck dissection. 6 Hospital returns among this vulnerable population may impact survival rates and expose patients to hospital-acquired complications. 5 Studies 1 7 8 9 10 have shown that between 9% and 59% of all unexpected readmissions may be prevented, and recognizing the causes is crucial to lowering the rates of unplanned returns and the corresponding healthcare expenses.

Previous studies 4 11 12 13 have identified rates of unplanned hospital returns after HNS ranging from 7.3% to 26.5%. A retrospective study 11 showed a rate of returns to the emergency department (ED) of 8.43%, with infections being the most common cause for returns to the hospital (26.8%). Another report 5 described wound complications as the most frequent cause of readmission (15.3%).

The causes for unplanned hospital return following HNS have yet to be clearly described within the Saudi population. We aim to identify the rate and causes of unplanned hospital returns and readmission within 60 days following HNS at a tertiary care center in Saudi Arabia. Addressing preventable causes may be beneficial in lowering the revisit rates.

## Methods

After obtaining ethical approval from the Institutional Review Board (IRB; reference number: 559–22), we reviewed the charts of patients who returned to the hospital through the ED or the outpatient patient department (OPD) within 60 days after HNS discharge between January 2015 and August 2022. We excluded all patients with missing data, such as those with no documented cause for hospital return. All patients were aged ≥ 18 years.

The primary outcome was to describe the causes of 60-day unplanned return through the ED or OPD, obtained as the final diagnosis from the hospital's record system. Only the first episode was extracted if more than one episode of unplanned returns was identified. The secondary outcome was to identify the rate of readmission as inpatients in those who returned.

We collected the medical record number, as well a data regarding age, gender, body mass index (BMI), and smoking status. Moreover, the documented primary site of surgery, the type of condition, whether benign or malignant, and the dates of primary admission, procedure, discharge, and return were also collected. The cases were classified into categories based on the procedure performed. Table 1 shows examples of procedures performed through these categories. We excluded procedures involving ears, tonsils, adenoids, or the skin. Moreover, robotic surgeries were not included in the study.

**Table 1:** Procedure categories included in the present study

Category	Procedures
Salivary gland	Parotidectomy Submandibular gland excision Sublingual gland excision Minor salivary gland surgery
Thyroid/parathyroid	Total thyroidectomy Hemithyroidectomy Parathyroidectomy
Sinonasal/skull base	Endoscopic resection of nasal neoplasms
Limited neck	Branchial cleft cyst excision Sistrunk procedure
Neck dissection only	Cervical lymph node dissection
Major head and neck with no flap	Laryngectomy without flap reconstruction Oropharyngeal resection without flap reconstruction
Major head and neck with pedicled flap	Resection of the oropharynx with reconstruction of pectoralis major myocutaneous rotation flap
Major head and neck with free flap	Oropharyngeal resection with forearm free flap reconstruction
Open airway	Tracheostomy
Limited oral cavity	Glossectomy (total or partial) Mandibulectomy (total or partial) Maxillectomy

Additionally, the comorbidities of the patients were obtained and evaluated using the American Society of Anesthesiologists (ASA) score and the Cumulative Illness Rating Scale (CIRS), a comorbidity scale that quantifies the overall disease burden through 13 relatively independent body systems. 14

### Statistical Analysis

Data were entered into Google Forms (Google, Mountain View, CA, United States) and then exported to Microsoft Excel, version 16.0 (Microsoft Corp., Redmond, WA, United States). The statistical analysis was performed using the IBM SPSS Statistics for Windows, version 21.0 (IBM Corp., Armonk, NY, United States), and statistical significance was set as *p* < 0.05 for all tests. Depending on the distribution, continuous variables were expressed as mean ± standard deviation (SD) or median and interquartile range (IQR) valuers. The categorical variables were expressed as numbers and frequencies. The means were compared using the Student *t* -test, the medians were compared using the Mann-Whitney U test, and the Chi-squared test was used to compare the frequencies. Variables with significant relationships in the univariate analysis were employed in the multivariate analysis.

## Results

In total, 1,030 patients underwent HNS at our center between 2015 and 2022; 119 (11.55%) returned to the hospital within 60 days after discharge, 19 of whom (1.84%) were readmitted as inpatients. Overall, 90 (8.74%) patients returned to the OPD, but only 9 (0.87%) were readmitted as inpatients. On the other hand, 29 (2.82%) patients returned to the ED, and 10 of them (0.97%) were readmitted. Table 2 describes the baseline characteristics and demographic data of the patients.

**Table 2:** Baseline characteristics and demographic data of the patients (n = 119)

Variable	
Age (in years): mean ± SD	49.76 ± 14.98
Female gender: n; %	74; 62.20%
Length of primary stay (in days): median (IQR)	4 (2–9)
CIRS score: mean ± SD	4.10 ± 2.83
ASA score: mean ± SD	2.12 ± 0.70
Previous radiotherapy: n; %	29; 24.4%
Previous chemotherapy: n; %	6; 5%
Previous chemoradiation therapy: n; %	5; 4.2%
Current smoker: n; %	5; 4.2%
Former smoker: n; %	5; 4.2%

Abbreviations: ASA, American Society of Anesthesiologists; CIRS, Cumulative Illness Rating Scale; IQR, interquartile range; SD, standard deviation.

As shown in Table 3 , the most frequent cause of OPD return was hematoma (20%). For ED returns, the causes are summarized in Table 4 . The most common cause for ED visits was neurological symptoms (20.69%), such as seizures, weakness, and numbness. Infections, including surgical site infection, oral thrush, and urinary tract infection (UTI), were the most common cause of readmission as an inpatient (26.32%). The rest of the causes for readmission as inpatients are summarized in Table 5 .

**Table 3:** Rates and causes of Outpatient Department visits

Causes	n	%
Hematoma	18	20
Neurological: seizure, weakness, peripheral numbness	13	14.44
Infections: surgical site, urinary tract infection, oral thrush	10	11.11
Pain in the surgical site	7	7.78
Respiratory: dyspnea, wheezing	7	7.78
Gastrointestinal: nausea, vomiting	6	6.67
Hoarseness	6	6.67
Fatigue	5	5.56
Equipment issues: tracheostomy, surgical drain	4	4.44
Facial nerve paralysis	4	4.44
Cardiac: chest pain, palpitation	3	3.33
Surgical site bleeding	3	3.33
Fistula	1	1.11

**Table 4:** Rates and causes of Emergency Department visits

Causes	n	%
Neurological: seizure, weakness, peripheral numbness	6	20.69
Equipment issues: tracheostomy, surgical drain	5	17.24
Infections: surgical site, urinary tract infection, oral thrush	4	13.79
Gastrointestinal: nausea, vomiting	3	10.34
Hematoma	3	10.34
Surgical site bleeding	3	10.34
Wound dehiscence	2	6.90
Cardiac: chest pain, palpitation	1	3.44
Psychiatric: delirium	1	3.44
Respiratory: dyspnea, wheezing	1	3.44

**Table 5:** Rates and causes of readmission as an inpatient

Causes	n	%
Infections: surgical site, urinary tract infection, oral thrush	5	26.32
Neurological: seizure, weakness, peripheral numbness	4	21.05
Equipment issues: tracheostomy, surgical drain	3	15.79
Gastrointestinal: nausea, vomiting	2	10.53
Wound dehiscence	2	10.53
Cardiac: chest pain, palpitation	2	10.53
Psychiatric: delirium	1	5.26

Table 6 compares the ED and OPD groups. We found that male patients were more likely to return to the ED than females (58.62% versus 41.38% respectively; *p* = 0.015). Additionally, ED patients had a significantly higher mean age than those who visited the OPD (54.72 versus 48.16 respectively; *p* = 0.039). Furthermore, malignancy as an indication for surgery was associated with ED returns ( *p* = 0.005). Patients who returned to the ED presented higher readmission rates as inpatients ( *p* = 0.005). Moreover, patients who visited the ED presented significantly higher ASA ( *p* = 0.01) and CIRS scores ( *p* = 0.005) than those who visited the OPD.

**Table 6:** Comparison between ED and OPD groups in gender, age, BMI, type of condition, rate of readmission as inpatients, ASA score, and CIRS score

Variable	ED	OPD	*p-* value
Gender: n (%)			
Male	17 (58.62)	28 (31.11)	0.015
Female	12 (41.38)	62 (68.89)	
Age (in years): mean ± SD	54.72 ± 18.06	48.16 ± 13.57	0.039
BMI (in Kg/m ^2^ ): n (%)			
< 18.5	4 (13.8)	8 (8.9)	0.679
18.5–24.9	5 (17.2)	15 (16.7)	
25–29.9	11 (37.9)	29 (32.2)	
30–34.9	6 (20.7)	17 (18.9)	
35–39.9	1 (3.4)	13 (14.4)	
≥ 40	2 (6.9)	8 (8.9)	
Type of condition: n (%)			
Benign	2 (6.9)	33 (36.7)	0.005
Malignant	27 (93.1)	57 (63.3)	
Readmission as inpatient: n (%)			
Yes	10 (34.5)	9 (10)	0.005
No	19 (65.5)	81 (90)	
ASA score: mean ± SD	2.41 ± 0.68	2.02 ± 0.69	0.01
CIRS score: mean ± SD	5.48 ± 2.97	3.66 ± 2.64	0.005

Abbreviations: ASA, American Society of Anesthesiologists; BMI, body mass index; CIRS, Cumulative Illness Rating Scale; ED, Emergency Department; OPD, Outpatient Department; SD, standard deviation.

Similarly, the mean age among the readmitted patients (57.95 ± 14.95 years) was significantly higher than that of nonreadmitted patients (48.20 ± 14.54 years) ( *p* = 0.009). Furthermore, malignant cases were more likely to be readmitted ( *p* = 0.025). The mean ASA score of readmitted patients (2.53 ± 0.61) was significantly higher than that of the subjects not readmitted as inpatients (2.04 ± 0.70) ( *p* = 0.004). Additionally, the mean CIRS score of the readmitted patients (6.26 ± 3.11) was higher than that of the subjects not readmitted as inpatients (3.69 ± 2.59) ( *p* = 0.002). There was a statistically significant positive correlation between the ASA and CIRS comorbidity scores when using simple linear regression ( *p* < 0.001), with r ^2^ = 0.301. A comparison between readmitted and nonreadmitted patients is shown in Table 7 .

**Table 7:** Comparison between readmitted and nonreadmitted patients regarding gender, age, BMI, type of condition, ASA score, and CIRS score

Variable	Readmitted	Nonreadmitted	*p-* value
Gender: n (%)			
Male	8 (42.1)	37 (37)	0.871
Female	11 (57.9)	63 (63)	
Age (in years): mean ± SD	57.95 ± 14.95	48.20 ± 14.54	0.009
BMI (in Kg/m ^2^ ): n (%)			
< 18.5	2 (10.5)	10 (10)	0.712
18.5–24.9	4 (21.1)	16 (16)	
25–29.9	5 (26.3)	35 (35)	
30–34.9	4 (21.1)	19 (19)	
35–39.9	1 (5.3)	13 (13)	
≥ 40	3 (15.8)	7 (7)	
Type of condition: n (%)			
Benign	1 (5.3)	34 (34)	0.025
Malignant	18 (94.7)	66 (66)	
ASA score: mean ± SD	2.53 ± 0.61	2.04 ± 0.70	0.004
CIRS score: mean ± SD	6.26 ± 3.11	3.69 ± 2.59	0.002

Abbreviations: ASA, American Society of Anesthesiologists; BMI, body mass index; CIRS, Cumulative Illness Rating Scale; SD, standard deviation.

The multivariate logistic regression analysis revealed significant risk factors for readmission after hospital discharge, including older age (odds ratio [OR] = 1.1; 95% confidence interval [95%CI]: 0.89–1.31; *p* = 0.003), malignant cases (OR = 0.29; 95%CI: 0.066–0.234; *p* = 0.011), higher ASA score (OR = 0.49; 95%CI: 0.19–0.82; *p* = 0.005), and higher CIRS score (OR = 0.44; 95%CI: 0.21–0.66; *p* = 0.029).

## Discussion

The rate of unplanned hospital returns following HNS was of 11.55%, and 1.84% of tese subjects were readmitted as inpatients; this is below the 3.2% to 14.5% readmission rates reported in other studies. 5 11 15 16 17 Bur et al. 15 studied the rate and predictive factors for readmission after HNS for malignant conditions and found a rate of 5.1% of readmissions as inpatients. Goel et al. 5 reported a rate of unplanned hospital readmission after sinonasal cancer surgery of 11.6%. The fact that we incorporated benign causes and malignant indications for HNS can explain the decreased readmission rates found in the present study. However, our study showed results similar to those of other studies 5 15 regarding the causes for readmission, with infections being the most common. Such etiologies may be preventable with proper patient and caregiver education. Although the specific antibiotic regimens and sterile procedures employed by different practitioners can vary significantly, antibiotic prophylaxis helps lower the occurrence of infection. 18 Cancer patients are particularly exposed to infections, and aggressive prophylactic treatment for head and neck cancer patients should gain more attention. This intervention may lower the rate of unplanned hospital returns, as most of the returned patients presented malignancies as an indication for HNS.

The rate of ED revisits after HNS has been described in the literature. Wu and Hall 11 reported an ED revisit rate of 8.43%, with pain being the most frequent reason. Another study 19 reported a rate of 11.22% of ED revisits following thyroidectomy and parathyroidectomy, with frequent causes being wound complications and paresthesia. In the present study, the rate of ED revisits after HNS was of 2.82%, with common causes being neurological symptoms, such as weakness, paresthesia, and seizures, as well as equipment issues, such as tracheostomy and surgical drain displacement. Early discharge planning, medication review on a case-by-case basis, and caregiver education about the importance of staying hydrated, as well as red flags for electrolyte abnormalities, may reduce ED returns. 20 21 Since surgical equipment problems are a common cause of hospital return, discharged patients with tracheostomies and surgical drains may benefit from earlier follow-up times. Online communication technologies are a potential solution for earlier, more frequent follow-ups, especially for those patients who live in peripheral areas and may need help with persistent follow-ups because of transportation issues and the referral process. Previous studies 22 23 emphasized the effectiveness of remote communication methods for earlier follow-ups in improving patient outcomes and decreasing unplanned hospital return rates. The Re-Engineered Discharge (RED) project employs pharmacists to contact patients by telephone two to four days after discharge to address questions and avert medication-related issues. 24

Previous reports 11 25 confirmed that the ASA score is closely linked to the prediction of readmissions and is positively associated with increased readmission rates. Moreover, the CIRS comorbidity score has been used in patients undergoing HNS, with higher scores indicating deteriorating baseline health. 11 14 26 Thus, it is believed that the patients readmitted in the present study had a higher baseline health burden, which left them exposed to more severe complications, leading to readmission as inpatients. Additionally, head and neck cancer patients present more comorbidities, frequently due to long-term exposure to risk factors, including alcohol and tobacco use. 27 28 29 This explains the findings of the present study, as most readmitted patients presented malignancy as an indication for HNS. More frequent and close postoperative follow-ups for patients with increased baseline health burdens may decrease the unplanned hospital readmission rate.

By extending the analysis period to 60 days rather than the usual 30 days after surgery, we provide exclusive and unique data about the reasons for unplanned hospital returns and ED use. The present study was conducted in a tertiary referral center in western Saudi Arabia; many cases are referred to our hospital from peripheral areas, and transportation and referral may compromise early follow-ups. Hence, extending the study period to 60 days after discharge may provide us with a bigger picture of the actual rate for unplanned hospital return after HNS. Nevertheless, our findings are to be interpreted with several limitations in mind. The typical challenge for retrospective studies is obtaining accurate and conclusive data about the exact surgical steps, cause and time for hospital return after discharge. Additionally, many nonmodifiable factors, such as age and socioeconomic status, as well as other factors unrelated to the surgery, may affect the unplanned hospital return rate within the first 60 days. Moreover, the generalizability of our findings may be constrained by the fact that our research was limited to a single center. Thus, more multicentric prospective studies with larger populations are warranted.

## Conclusion

The rate of unplanned hospital return within 60 days was of 11.55% (8.74% through the OPD and 2.82% through the ED), and 1.84% of these patients were readmitted. Hematoma, infections, and neurological symptoms were common causes. Addressing common reasons may serve as a step in lowering hospital return and readmission rates. Similar data may be used to design interventions that may be beneficial to decrease the unplanned hospital return rate.
